# Comment on: Prediction and secondary prevention of preeclampsia from the perspective of public health management – the initiative of the State of Rio de Janeiro

**DOI:** 10.61622/rbgo/2024rbgo80

**Published:** 2024-08-02

**Authors:** Karina Bilda de Castro Rezende, Danielle Sodré Barmpas, Heron Werner, Daniel Lorber Rolnik, Fabricio da Silva Costa, Liona Poon, Marianna Amaral Pedroso, Lisandra Stein Bernardes, Juliana Costa Rezende, Conrado Sávio Ragazini, Rita Guérios Bornia, David Wright, Kypros Nicolaides, Cristos Pritsivelis, Renato Augusto Moreira de Sá

**Affiliations:** 1 Universidade Federal do Rio de Janeiro Maternity School Rio de Janeiro RJ Brazil Maternity School, Universidade Federal do Rio de Janeiro, Rio de Janeiro, RJ, Brazil.; 2 Fundação Oswaldo Cruz Instituto Fernandes Figueira Rio de Janeiro RJ Brazil Instituto Fernandes Figueira, Fundação Oswaldo Cruz, Rio de Janeiro, RJ, Brazil.; 3 Pontifícia Universidade Católica do Rio de Janeiro Department of Fetal Medicine Rio de Janeiro RJ Brazil Department of Fetal Medicine, Pontifícia Universidade Católica do Rio de Janeiro, Rio de Janeiro, RJ, Brazil.; 4 Monash University Department of Obstetrics and Gynaecology Melbourne Australia Department of Obstetrics and Gynaecology, Monash University, Melbourne, Australia.; 5 Griffith University Gold Coast University Hospital and School of Medicine and Dentistry Maternal Fetal Medicine Unit Gold Coast Australia Maternal Fetal Medicine Unit, Gold Coast University Hospital and School of Medicine and Dentistry, Griffith University, Gold Coast, Australia.; 6 The Chinese University of Hong Kong Department of Obstetrics and Gynecology Hong Kong China Department of Obstetrics and Gynecology, The Chinese University of Hong Kong, Hong Kong.; 7 Maternal Fetal Medicine Unit Belo Horizonte MG Brazil Maternal Fetal Medicine Unit, Oncoclínicas, Belo Horizonte, MG, Brazil.; 8 Aalborg University North Denmark Regional Hospital Obstetrics and Gynecology Department Denmark Centre for Clinical Research, Obstetrics and Gynecology Department, North Denmark Regional Hospital, Aalborg University, Denmark.; 9 Universidade de Brasília Department of Obstetrics and Gynecology Brasília DF Brazil Department of Obstetrics and Gynecology, Universidade de Brasília, Brasília, DF, Brazil.; 10 Universidade de São Paulo Faculdade de Medicina Ribeirão Preto SP Brazil Faculdade de Medicina, Universidade de São Paulo, Ribeirão Preto, SP, Brazil.; 11 University of Exeter Institute of Health Research Exeter UK Institute of Health Research, University of Exeter, Exeter, UK.; 12 King's College Hospital Fetal Medicine Research Institute London UK Fetal Medicine Research Institute, King's College Hospital, London, UK.; 13 Universidade Federal de Fluminense Department of Obstetrics and Gynaecology Rio de Janeiro RJ Brazil Department of Obstetrics and Gynaecology, Universidade Federal de Fluminense, Rio de Janeiro, RJ, Brazil.

Dear Editor,

We read with interest the article "Prediction and secondary prevention of preeclampsia from the perspective of public health management—the initiative of the State of Rio de Janeiro," published by Braga and colleagues.^(1)^ The authors recommend universal treatment (for all pregnant women) with elemental calcium at 1500 mg per day and identification of women at high-risk of preeclampsia (PE) based on the presence or absence of maternal risk factors alone, followed by aspirin at a dose of 100 mg in those cases. In this letter, we express our concerns with these simplistic strategies which, despite best intentions, are unlikely to work in clinical practice or reduce the PE rates at the population level.

First, the evidence that 1500 mg of elemental calcium supplementation is useful in preventing preeclampsia (PE) is, at best, questionable. There is no evidence to support universal use of calcium, or cost-effectiveness data supporting such universal treatment strategy. It is true that the World Health Organization (WHO) guideline^(2)^ issued in 2011 and updated in 2018,^(2,3)^ supported by Cochrane Systematic Reviews,^(4,5)^ recommended calcium supplementation during pregnancy in areas with low dietary calcium intake. However, a sensitivity analysis evaluated the validity of these Cochrane systematic reviews and meta-analyses,^(6)^ and found that conclusions about the benefits of calcium supplementation to prevent PE are misleading because of a high degree of heterogeneity (I^2^ = 76%) between trials, with results mainly driven by a large number of small studies included in the metanalysis. Small studies tend to overestimate treatment effects ("small-study effects") and are more likely to be published when positive findings are reported than when no treatment effect is evident (publication bias).^(7)^ Of note, the three larger randomized trials of calcium for the prevention of PE reported negative findings and had no heterogeneity when combined.^(6)^

Second, pregnant women are naturally resistant to medication use due to concerns regarding safety, risk perception, and effectiveness^(8)^ that can be aggravated by non-convincing medical advice. Considering this suboptimal medication adherence during pregnancy, incorporating a complex dose scheme of 1500 mg of calcium supplement per day can also jeopardize dietary nonheme iron and iron absorption^(9)^ and interfere with compliance to aspirin indicated for pregnant women classified as high-risk of developing PE.

Although the risk of preterm PE can be reduced by the prophylactic use of aspirin with a daily dose ≥ 100 mg and starting the therapy < 16 weeks,^(10)^ the ASPRE trial^(11)^ utilized 150 mg of aspirin per day, based on previous evidence showing a dose-dependent benefit of the therapy,^(12)^ and achieved a 62% reduction in the risk of PE. Thus, we agree that it would be ideal for the pharmaceutical industry to commercialize this dosage, as Braga et al.^(1)^ suggested. However, until this is achieved, a dose of 150mg of aspirin can be easily achieved by administering 1 + 1/2 tablet of 100 mg and discarding the other half.^(13)^

Last but not least, the proposed risk factor checklist approach is known to perform poorly, detecting less than a third of patients who will later develop PE.^(14)^ The most effective way of identifying the high-risk group to receive prophylactic aspirin is by combining maternal factors with biophysical and biochemical markers,^(15)^ as used in the ASPRE trial employing an algorithm freely provided by Fetal Medicine Foundation (FMF),^(16)^ through software, website, and mobile device applications with a user-friendly interface available at https://fetalmedicine.org/research/assess/preeclampsia.^(16,17)^ The most significant components of combined screening, such as mean arterial pressure measurement and ultrasound, are readily available and widely used in most clinical settings, even in low/middle-income countries such as Brazil, and do not incur additional costs. Even without biochemical markers, the FMF algorithm outperforms risk factor-based checklists and could be rapidly implemented, leading to a significant increase in the identification of high-risk women who would benefit from aspirin prophylaxis. More than half of these women would likely be missed by the traditional risk factor-based approach.^(18)^

The Rio de Janeiro State Secretariat for Health misses a critical window of opportunity to effectively mitigate the burden of PE by not considering implementing a feasible universal PE screening program with effective prophylactic measures based on the screening result, in line with recent recommendations from the International Federation of Gynecology and Obstetrics (FIGO),^(13)^ the International Society for the Study of Hypertension in Pregnancy (ISSHP),^(19)^ and the International Society of Ultrasound in Obstetrics and Gynecology (ISUOG).^(20)^ The FMF algorithm with biophysical markers and without using biochemical markers has been previously validated in Rio de Janeiro and achieved a detection rate of 71%,^(21)^ similar to the reference study.^(22)^ A pragmatic FIGO recommendation is to use a combination of maternal risk factors with mean arterial pressure as a baseline screening test to estimate each individual's risk of developing preterm PE, where it is not possible to measure uterine artery pulsatility index and biochemical markers. It is better to use this simple combined test rather than using maternal risk factors as binary (yes/no) variables alone, as the latter approach leads to suboptimal prediction.^(13)^ In light of these considerations, we advise that all pregnant women should be screened for preterm PE during early pregnancy by the FMF algorithm, women identified at high risk should receive aspirin prophylaxis at a dose of 150 mg and, calcium supplementation should be reserved for women at high-risk for PE and low dietary calcium intake, rather than universally.
