# Supplementary material for: Comment on: Prediction and secondary prevention of preeclampsia from the perspective of public health management – the initiative of the State of Rio de Janeiro
Source: Rev Bras Ginecol Obstet. 2024 Aug 2;46:e-rbgo80. doi: 10.61622/rbgo/2024rbgo80 (PMC11341189; doi:10.61622/rbgo/2024rbgo80)
Supplement: Related texts

# Author's reply to Comment on: Prediction and secondary prevention of preeclampsia from the perspective of public health management – the initiative of the State of Rio de Janeiro

**Authors:** Antonio Braga, Penélope Saldanha Marinho, Marcos Nakamura-Pereira, José Carlos Peraçoli, Cláudia Mello

**Affiliations:** 1 Universidade Federal Fluminense Niterói RJ Brazil Universidade Federal Fluminense, Niterói, RJ, Brazil.; 2 Universidade de Vassouras Vassouras RJ Brazil Universidade de Vassouras, Vassouras, RJ, Brazil.; 3 Secretária de Estado de Saúde do Rio de Janeiro Rio de Janeiro RJ Brazil Secretária de Estado de Saúde do Rio de Janeiro, Rio de Janeiro, RJ, Brazil.; 4 Universidade Federal do Rio de Janeiro Rio de Janeiro RJ Brazil Universidade Federal do Rio de Janeiro, Rio de Janeiro, RJ, Brazil.; 5 Fundação Oswaldo Cruz Instituto Nacional de Saúde da Mulher, da Criança e do Adolescente Fernandes Figueira Rio de Janeiro RJ Brazil Instituto Nacional de Saúde da Mulher, da Criança e do Adolescente Fernandes Figueira, Fundação Oswaldo Cruz, Rio de Janeiro, RJ, Brazil.; 6 Faculdade de Medicina de Botucatu Departamento de Obstetrícia e Ginecologia Botucatu SP Brazil Departamento de Obstetrícia e Ginecologia, Faculdade de Medicina de Botucatu, Botucatu, SP, Brazil.

Dear Editor,

We thank the authors Rezende et al.^(1)^ the opportunity to continue discussing the preeclampsia prevention strategies adopted by the State of Rio de Janeiro and which was the reason for our editorial in RBGO.^(2)^

The first of the criticisms targeted the World Health Organization's (WHO) recommendation^(3)^ for universal calcium supplementation for pregnant women from areas with low intake.^(4)^ There were two questions raised on this topic.

The first of them would be that taking 3 calcium tablets a day (for a total of 1500mg) could be a complex scheme, with potential harm to iron absorption or even adherence to acetylsalicylic acid (AAS) when necessary. We agree that calcium supplementation can impair iron absorption and, therefore, we advise in our Editorial that calcium tablets should be ingested with some food, but not those rich in phytates, oxalates, or iron, as these substances hinder the calcium absorption.^(2)^ Furthermore, calcium supplements should be taken at least 2 hours apart from iron supplements or multivitamins containing iron, as this mineral reduces calcium absorption.^(2)^ We believe that, with this guidance, and the correct encouragement to adhere to this supplementation, coordinated by the prenatal care provider, showing that calcium is not a medicine, but a supplement, just like iron and folic acid, whose supplementation is well accepted among our pregnant women, that these issues will be resolved.

The other question concerns the effectiveness of preventing preeclampsia with calcium supplementation in populations with low intake. The authors cite Wright's et al.^(4)^ study that analyzed Hofmeyr's et al.^(5)^ meta-analysis which supported the WHO recommendation^(3)^ regarding universal calcium supplementation during pregnancy in areas with low intake. Wright's et al.^(4)^ analysis evaluating the heterogeneity of the studies included in the meta-analysis had already been presented by Hofmeyr et al.^(5)^ in the original study. In response to Wright's et al.^(4)^ questions, the authors of the meta-analysis emphasized that: "The composite outcome maternal death or serious morbidity was reduced with calcium supplementation (four trials, 9732 women; RR: 0.80, 95% CI: 0.66–0.98). There was one maternal death with calcium supplementation versus six maternal deaths with placebo. These important outcomes were not subject to heterogeneity or small trial effects".^(6)^ We are convinced that our main target in this action to prevent preeclampsia in the State of Rio de Janeiro is to reduce maternal mortality. And the data points in our favor. Furthermore, we can not lose sight of the fact that hypertension (all forms) is the main cause of maternal death in the state of Rio de Janeiro and we have evidence that the incidence of hypertension during pregnancy affect 12% of women^(7)^ and seems higher in Brazil than in other countries. Therefore, any reduction in the occurrence of this problem is of special interest to public health.

We are certainly looking forward to the inclusion of two recent simultaneous trials in this meta-analysis, as their results reinforce the benefits of universal calcium supplementation during pregnancy in populations with low intake, even at lower doses.^(8)^ We hope the WHO can reduce its supplementation recommendation from 1500-2000mg of daily calcium to 500mg (that would certainly facilitate adherence to supplementation), which seems to provide the same protection against the ominous outcomes of preeclampsia. Until then, we are staunch that following the WHO does not seem unreasonable.

The authors’ other relevant question concerns the use of AAS in preventing preeclampsia. And then there are also two issues that were raised: the dose of AAS and the type of screening used to predict it.

Regarding the dose of AAS, the authors advocate the advantages of using 150mg compared to the 100mg that we propose, according to the Brazilian Ministry of Health^(9)^ and the Brazilian Network for the Study of Hypertension in Pregnancy (Rede Brasileira de Estudos de Hipertensão na Gestação – RBEHG).^(10)^ It is worth highlighting that the majority of the 77 trials included in the Cochrane review that evaluated the role of antiplatelet agents for preventing pre-eclampsia and its complications used low doses of AAS.^(11)^ Only one trial with 1,776 women used 150mg of AAS in this metanalysis. Thus, the results of this Cochrane review demonstrated the effectiveness of prophylaxis with 100mg of AAS not only in consistently reducing preeclampsia, but also in the occurrence of preterm birth, infant deaths, neonatal deaths or death before hospital discharge, and other adverse outcomes.^(11)^

The benefits of using higher doses of AAS were validated by the ASPRE study,^(12)^ and led to a greater response in preventing preeclampsia. Specifically regarding the AAS dose of 150mg, it is worth noting that Brazil does not sell AAS in this dosage. As organizers of care, state public managers need to anchor their policies by prioritizing medicines included in the Brazilian National List of Essential Medicines (*Relação Nacional de Medicamentos Essenciais – RENAME*).^(13)^ And a simple consultation of this list shows that AAS is available through the Brazilian Unified Health System (Sistema Único de Saúde – SUS) in doses of 100 and 500mg. The authors point to two solutions to this problem that bring major concern. The first of these is the instruction to break the tablets in order to obtain a dosage of 150mg. The Brazilian National Health Surveillance Agency (*Agência Nacional de Vigilância Sanitária*) does not regulate this practice^(14)^ and does not require that any grooves in the tablets be related to the division of the active ingredient into halves of the product.^(15)^ Of special relevance in the case of AAS is the protection that certain tablets of this product have to avoid gastric action, which is lost when the tablet is split. Furthermore, pharmaceutical companies do not recommend this practice and the responsibility for any error in the dosage administered will be attributed to the professional who advised this practice.^(15)^ The authors’ other recommendation would be to discard the other half of the AAS tablet not immediately used, which would lead to a loss of 25% of all AAS used in an eventual government preeclampsia prevention program. Regarding the fiscal responsibility required of public managers, this policy does not meet the rigors of public pharmacoeconomics and would probably be rejected by control agencies and the public ministry itself. Finally, in relation to the dose of AAS, it is also worth highlighting the WHO's concern with the lack of evidence on the comparative risk of postpartum haemorrhage among women who received 75mg (dose recommended by WHO to preeclampsia prevention) compared with those who received 150mg of aspirin and noted it as a research priority.^(16)^

Regarding organized screening for the prediction of preeclampsia, we agree with the authors about its importance, including the use of biochemical and biophysical markers for this. However, budgetary limitations prevent this strategy from being implemented at this moment. Even among Brazilian university centers, which serve a tiny portion of the obstetric population, we do not know of any service that performs this screening in its routine. Even in well-resourced clinical settings, as highlighted by Magee et al.,^(17)^ uterine-artery Doppler ultrasonography and placental growth factor assays are not routinely performed. In addition to the costs of biochemical markers, the use of uterine-artery Doppler ultrasonography implies the availability of this test in the SUS routine, which has not yet been validated by the Brazilian Ministry of Health. Considering that a significant proportion of pregnant women in the State of Rio de Janeiro begin prenatal care at more than 12 weeks of gestation, the imposition of biophysical variables for the onset of AAS, which would certainly take time, would postpone the start of prophylaxis for preeclampsia beyond the 16th weeks of pregnancy, which could reduce its effectiveness. Even the use of just mean arterial pressure, something clinically simple to do, as a strategy also recommended by the International Federation of Gynecology and Obstetrics (FIGO),^(18)^ encounters some limitations, including the lack of computers/internet networks, especially in rural areas of 92 municipalities that make up the State of Rio de Janeiro. However, adding mean arterial pressure to maternal risk factors increases the detection rate of preeclampsia before the 37^th^ weeks of gestation from 41.5% (33.3-50.1) to 49.3% (40.8-57.8),^(19)^ which we consider important. Therefore, we predict that this strategy could be the next target we should pursue.

Organizing the prediction of preeclampsia in the State of Rio de Janeiro considering only clinical factors, in accordance with the recommendations of the American College of Obstetricians and Gynecologists (ACOG), the Society for Maternal-Fetal Medicine (SMFM), the U.S. Preventive Services Task Force (USPSTF)^(20)^ and the RBEHG^(10)^ do not seem like a missed window of opportunity. Considering that calcium is not supplemented for almost no pregnant woman in the State of Rio de Janeiro and the use of AAS is extremely limited, even among patients with unequivocal clinical risk factors for preeclampsia, organize the obstetric care network with the introduction of prediction model and actions of preeclampsia prevention with universal physical activity and calcium supplementation, as well as AAS in cases selected based on clinical variables, will pave the way for more refined strategies that, we hope, will not be delayed. In public management, science must go hand in hand with wisdom. Or as St. Francis of Assisi would say: "Start by doing what is necessary, then what is possible, and suddenly you are doing the impossible!".

For now, and while the universal use of biophysical and biochemical markers in screening for preeclampsia, as recommended in ASPRE, is not possible, we consider that universal calcium supplementation for pregnant women in the State of Rio de Janeiro, whose diet is known to be deficient in this mineral^(21)^ as well as 100mg of AAS prophylaxis carried out in those cases of clinical risk factors obtained during the first prenatal consultation seems to be appropriate for our obstetric population.^(2)^ Considering the high rates of maternal mortality in our State, led by hypertension during pregnancy/preeclampsia/eclampsia, the impossibility of offering a screening capable of predicting preeclampsia, with greater precision, should not invalidate the policy now established. As Voltaire said: "the best is the enemy of good". Strengthening the line of care for pregnant women, especially universalizing the prediction of preeclampsia through organized screening, even if only with clinical factors, is a valuable initiative and one that we consider worthy. A more precise guideline, but not applicable in clinical practice (for whatever reason) is worth less than a less comprehensive protocol but widely and immediately implementable in the obstetric routine of the SUS. After all, we are left with Confucius’ maxim "better a diamond with a flaw than a pebble without".

## References

[1] Rezende KB, Barmpas DS, Werner H, Rolnik DL, Costa FS, Poon L, Braga A, Marinho PS, Nakamura-Pereira M, Peraçoli JC, Mello C (2024). Prediction and secondary prevention of preeclampsia from the perspective of public health management – the initiative of the State of Rio de Janeiro [letter]. Rev Bras Ginecol Obstet.

[2] Braga A, Marinho PS, Nakamura-Pereira M, Peraçoli JC, Mello C (2024). Prediction and secondary prevention of preeclampsia from the perspective of public health management – the initiative of the State of Rio de Janeiro. Rev Bras Ginecol Obstet.

[3] World Health Organization (2011). Recommendations for prevention and treatment of pre-eclampsia and eclampsia.

[4] Wright D, Wright A, Magee LA, von Dadelszen P, Nicolaides KH (2024). Calcium supplementation for the prevention of pre-eclampsia: challenging the evidence from meta-analyses. BJOG.

[5] Hofmeyr GJ, Lawrie TA, Atallah AN, Duley L, Torloni MR (2014). Calcium supplementation during pregnancy for preventing hypertensive disorders and related problems. Cochrane Database Syst Rev.

[6] Hofmeyr GJ, Atallah AN, Torloni MR (2024). Evidence for calcium in pregnancy: more than pre-eclampsia. BJOG.

[7] Nakamura-Pereira M, Esteves-Pereira AP, Dias MA, Maia LK, Pacheco VE, Viellas EF (2016). Maternal and neonatal outcomes associated with hypertensive disorders during pregnancy: data from "Birth in Brazil" study: medical complications of pregnancy related to hypertensive syndromes. Pregnancy Hypertens.

[8] Dwarkanath P, Muhihi A, Sudfeld CR, Wylie BJ, Wang M, Perumal N (2024). Two randomized trials of low-dose calcium supplementation in pregnancy. N Engl J Med.

[9] Ministério da Saúde (2022). Secretaria de Atenção Primária à Saúde. Departamento de Ações Programáticas. Manual de gestação de alto risco.

[10] Peraçoli JC, Costa ML, Cavalli RC, de Oliveira LG, Korkes HA, Ramos JG (2023). Pré-eclâmpsia – Protocolo 2023.

[11] Duley L, Meher S, Hunter KE, Seidler AL, Askie LM (2019). Antiplatelet agents for preventing pre-eclampsia and its complications. Cochrane Database Syst Rev.

[12] Rolnik DL, Wright D, Poon LCY, Syngelaki A, O’Gorman N, de Paco Matallana C (2017). ASPRE trial: performance of screening for preterm pre-eclampsia. Ultrasound Obstet Gynecol.

[13] Ministério da Saúde (2022). Relação Nacional de Medicamentos Essenciais Rename 2022.

[14] Ministério da Saúde (2023). Agência Nacional de Vigilância Sanitária. Resolução RDC No. 140, de 29 de maio de 2003.

[15] Teixeira MT, Sá-Barreto LC, Silva DL, Cunha-Filho SS (2016). Panorama dos aspectos regulatórios que norteiam a partição de comprimidos. Rev Panam Salud Publica.

[16] World Health Organization (2021). WHO recommendations on antiplatelet agents for the prevention of pre-eclampsia.

[17] Magee LA, Nicolaides KH, von Dadelszen P (2022). Preeclampsia. N Engl J Med.

[18] Poon LC, Shennan A, Hyett JA, Kapur A, Hadar E, Divakar H (2019). The International Federation of Gynecology and Obstetrics (FIGO) initiative on pre-eclampsia: a pragmatic guide for first-trimester screening and prevention. Int J Gynaecol Obstet.

[19] Tan MY, Wright D, Syngelaki A, Akolekar R, Cicero S, Janga D (2018). Comparison of diagnostic accuracy of early screening for pre-eclampsia by NICE guidelines and a method combining maternal factors and biomarkers: results of SPREE. Ultrasound Obstet Gynecol.

[20] LeFevre ML, U.S. Preventive Services Task Force (2014). Low-dose aspirin use for the prevention of morbidity and mortality from preeclampsia: U.S. Preventive Services Task Force recommendation statement. Ann Intern Med.

[21] Instituto Brasileiro de Geografia e Estatística (2011). Coordenação de Trabalho e Rendimento. Pesquisa de orçamentos familiares: 2008-2009.
